# Burnout and Moral Injury in Healthcare Workers: An Observational Study in a Romanian Chronic Care Hospital

**DOI:** 10.3390/healthcare13182278

**Published:** 2025-09-12

**Authors:** Enășoni Sorina, Szekely Diana, Raluca Mioara Cosoroabă, Flavia Zara, Dorin Novacescu, Cristina Stefania Dumitru, Raul Patrascu, Alexandra Enache

**Affiliations:** 1 Doctoral School, “Victor Babes” University of Medicine and Pharmacy Timisoara, E. Murgu Square, Nr. 2, 300041 Timisoara, Romania; sorina.enasoni@umft.ro (E.S.); diana.szekely@umft.ro (S.D.); 2 Faculty of Dental Medicine, “Victor Babes” University of Medicine and Pharmacy, Revolutiei Ave. 1989, Nr. 9, 300580 Timisoara, Romania; cosoroaba.raluca@umft.ro; 3 Department II of Microscopic Morphology, Discipline of Histology, “Victor Babes” University of Medicine and Pharmacy Timisoara, E. Murgu Square, Nr. 2, 300041 Timisoara, Romania; flavia.zara@umft.ro (F.Z.); novacescu.dorin@umft.ro (D.N.); cristina-stefania.dumitru@umft.ro (C.S.D.); 4 Department of Functional Sciences, “Victor Babes” University of Medicine and Pharmacy, 300041 Timisoara, Romania; 5 Department VIII, Discipline of Forensic Medicine, Bioethics, Deontology and Medical Law, “Victor Babes” University of Medicine and Pharmacy Timisoara, E. Murgu Square, Nr. 2, 300041 Timisoara, Romania; enache.alexandra@umft.ro; 6 Center for Ethics in Human Genetic Identifications, “Victor Babes” University of Medicine and Pharmacy Timisoara, E. Murgu Square, Nr. 2, 300041 Timisoara, Romania

**Keywords:** burnout, professional, moral injury, health personnel, chronic disease, hospitals, chronic, Romania

## Abstract

Background/Objectives: Healthcare workers in chronic care hospitals are vulnerable to psychosocial risks such as burnout and moral injury due to prolonged patient exposure and limited institutional support. This study assessed the prevalence of burnout and moral injury among staff at the Chronic Diseases Hospital of Sebiș, Romania, and examined their associations with perceived stress and managerial support. Methods: A cross-sectional study was conducted between October 2022 and October 2024, including 62 healthcare workers (physicians, nurses, and auxiliary staff). Participants completed a sociodemographic survey, the Maslach Burnout Inventory (MBI), the Moral Injury Symptom Scale-Health Professional (MISS-HP), and additional items on perceived stress and institutional support. Statistical analysis included descriptive statistics, group comparisons, correlation matrices, and logistic regression. Results: High emotional exhaustion (MBI-EE ≥ 27) was reported by 45.2% of participants, with the highest rates among nurses (50%) and auxiliary staff (45.5%). Mean moral injury scores were moderate (mean = 5.3), with elevated levels observed in nurses and auxiliary staff. Pearson correlation analysis revealed no strong linear associations between burnout dimensions and moral injury. Logistic regression did not identify emotional exhaustion, perceived stress, or support as significant predictors of high moral injury. Conclusions: Burnout and moral injury are prevalent but appear to be partially dissociated in this Romanian chronic care setting. Moral injury may arise from contextual ethical pressures beyond general occupational strain. Interventions should focus on ethical climate, institutional responsiveness, and peer-based moral support to enhance staff resilience.

## 1. Introduction

Burnout and moral injury have become critical concerns in modern healthcare systems, especially in settings with prolonged patient exposure and limited institutional resources, such as chronic care hospitals. Healthcare workers are increasingly exposed to high workloads, ethical dilemmas, insufficient organizational support, and emotionally charged decisions, all of which contribute to heightened psychosocial risks [1,2,3].

Burnout is a psychological syndrome resulting from chronic occupational stress, characterized by emotional exhaustion, depersonalization, and a reduced sense of personal accomplishment [4]. It has been shown to affect up to 50% of nurses and over 40% of physicians in various settings, with increased prevalence during the COVID-19 pandemic, particularly in long-term care units and among frontline workers [5,6]. Burnout affects mental health and job satisfaction and contributes to medical errors, poor outcomes, and staff turnover [7].

Moral injury, although initially defined in military contexts, is increasingly recognized in healthcare as a consequence of repeated exposure to ethically distressing situations. It occurs when individuals are unable to act in accordance with their moral beliefs due to institutional constraints, conflicting priorities, or resource scarcity [8,9]. Healthcare workers experiencing moral injury often report guilt, shame, helplessness, and professional disengagement [10]. While related to burnout, moral injury has distinct features and consequences, requiring targeted evaluation and interventions [11].

Despite growing international literature, limited research has explored the intersection between burnout and moral injury in Eastern European healthcare systems. In Romania, where public hospitals face chronic underfunding, staff shortages, and unclear ethical frameworks, chronic care institutions represent high-risk environments for both syndromes [12]. A better understanding of how these phenomena interact in under-resourced settings is needed to develop sustainable strategies for professional well-being and ethical resilience.

Recent studies have emphasized that burnout and moral injury are interrelated yet distinct constructs, shaped by both individual and systemic factors [13]. Burnout is typically associated with chronic occupational stress, while moral injury arises from perceived violations of deeply held ethical values in the context of constrained or conflicting professional duties [14]. For example, moral injury may occur in situations where healthcare workers must follow institutional policies that conflict with their moral judgment, such as resource rationing or restrictions on patient autonomy [1,8]. Furthermore, the syndromes may co-occur but diverge in terms of psychological impact and required interventions. Emerging research also highlights the mediating role of psychological flexibility and emotion regulation in modulating individual susceptibility to both burnout and moral distress [15]. In Eastern Europe, the small amount of research in this area, especially in chronic care environments, calls for urgent empirical attention [16]. Exploring these concepts in a Romanian setting provides a unique opportunity to examine how under-resourced health systems influence the ethical climate and staff well-being.

This study aims to assess the prevalence and correlation between burnout and moral injury among healthcare workers in a Romanian chronic care hospital, and to explore how these outcomes relate to institutional support and perceived ethical stress. The results may inform practical and ethical frameworks to mitigate occupational suffering and improve quality of care in long-term health institutions. The setting of a chronic care hospital was deliberately chosen because such settings are often characterized by prolonged patient exposure, ethical ambiguity, and low institutional visibility—conditions that intensify moral and emotional strain. Despite their relevance, these institutions remain underrepresented in research on moral harm, particularly in Eastern European healthcare systems.

### Theoretical Background

Burnout and moral injury are two distinct but often overlapping constructs that describe different responses to psychological and ethical stressors in healthcare environments. Burnout, as originally conceptualized by Maslach and colleagues, includes three core dimensions: emotional exhaustion, depersonalization, and reduced personal accomplishment [4]. It arises primarily from chronic work-related stress, high workloads, and insufficient recovery time, and is particularly prevalent in emotionally demanding settings such as chronic care units [5].

In the context of chronic care institutions, burnout and moral injury may coexist yet originate from different organizational pressures. Burnout typically results from sustained exposure to emotional demands, high workload, and low reward, while moral injury arises when healthcare workers are compelled to act against their ethical values or witness moral transgressions without institutional support [17]. Theoretically, both constructs share a foundation in occupational strain and value incongruence; however, burnout emphasizes emotional depletion, whereas moral injury focuses on ethical disruption and existential dissonance [17]. Some authors argue that moral injury can be mistakenly labeled as burnout when in fact it reflects deeper moral disillusionment [18,19]. Empirical data on their correlation are mixed: while some studies report moderate associations, others suggest weak or no correlation, especially in settings where ethical climate is a dominant factor [20]. In this study, we anticipated some degree of overlap but also conceptual divergence, especially given the hierarchical rigidity and resource limitations present in Romanian chronic care facilities.

Moral injury, although originally described in military populations, has increasingly gained recognition in healthcare settings. It refers to the psychological distress resulting from actions—or the lack of them—which violate one’s moral or ethical code [8]. In healthcare, this may manifest when clinicians are forced to act against their conscience due to institutional policies, limited resources, or hierarchical constraints [9]. Unlike burnout, moral injury is not reducible to emotional fatigue, but involves deeper ethical dissonance and moral disillusionment.

Recent frameworks suggest that while both phenomena can co-occur, they differ in their etiology, symptomatology, and required interventions [10,11]. Burnout tends to respond to workload reduction and resilience training, whereas moral injury may require ethical repair, institutional accountability, and value-based leadership [21]. Understanding their theoretical underpinnings is essential for designing appropriate support strategies and interpreting patterns in staff-reported distress.

Based on the existing literature and the organizational characteristics of chronic care settings, the following hypotheses were formulated:

- A substantial proportion of healthcare workers in a Romanian chronic care hospital will report high levels of emotional exhaustion and moral injury.
- There will be a positive correlation between burnout (as measured by emotional exhaustion) and moral injury scores.
- Perceived stress and low institutional support will be associated with higher levels of moral injury.
- Nurses and auxiliary staff will report higher levels of burnout and moral injury compared to physicians.

## 2. Materials and Methods

*Study Design*. This observational, cross-sectional study was conducted between October 2022 and October 2024 in the Chronic Diseases Hospital of Sebiș, Arad County, Romania. The research aimed to evaluate the prevalence and correlation of burnout and moral injury among healthcare professionals working in a chronic care setting, with particular focus on perceived ethical stressors and institutional support.

*Study Population*. The study included a convenience sample of 62 healthcare workers (nurses, physicians, auxiliary staff) who volunteered to participate and met the following inclusion and exclusion criteria:

Inclusion criteria:

- Active employment at the Chronic Diseases Hospital of Sebiș;
- At least one year of professional experience;
- Provided written informed consent.

Exclusion criteria:

- Medical or maternity leave during the data collection period;
- Refusal or inability to complete the questionnaire.

No formal power calculation was performed prior to data collection, as the study was designed as an exploratory observational analysis within a single institution, targeting the entire active clinical staff during the study period. Given the small size of the hospital and the limited number of eligible employees, a census-like recruitment strategy was adopted to include all available personnel who met the inclusion criteria.

Out of 68 eligible healthcare workers employed at the Chronic Diseases Hospital of Sebiș during the study period, 62 agreed to participate, yielding a response rate of 91.2%.

*Instruments and Measures*. Data were collected using a standardized assessment battery, which included:

- Sociodemographic questionnaire: age, gender, professional role, years of experience.
- Maslach Burnout Inventory—Human Services Survey (MBI-HSS): assessing Emotional Exhaustion (EE), Depersonalization (DP), and Personal Accomplishment (PA) [13].
- Moral Injury Symptoms Scale—Health Professional (MISS-HP): measuring symptoms of guilt, betrayal, loss of trust, and moral dissonance [11].
- Custom-designed items: evaluating perceived ethical conflict frequency and perceived managerial support on Likert scales from 1 (very low) to 5 (very high).

For the purposes of primary analysis and group comparisons, we focused on the Emotional Exhaustion (EE) subscale, as it is the most validated and widely used core dimension of burnout in healthcare research. Several large-scale studies have demonstrated that EE is the most predictive subcomponent for adverse outcomes such as reduced job satisfaction, absenteeism, and intention to leave the profession [22]. However, data for all three MBI dimensions (EE, DP, PA) were collected and were included in correlational analyses to capture the broader burnout profile. All instruments were administered in Romanian. Validated Romanian translations of the Maslach Burnout Inventory (MBI-HSS) and the Moral Injury Symptoms Scale–Health Professional (MISS-HP) were used, with additional items developed and reviewed in Romanian to ensure linguistic and contextual relevance.

To our knowledge, this is the first study to apply the MISS-HP scale in a Romanian healthcare context. The Romanian translation was carefully reviewed by the research team for semantic clarity and cultural relevance. During administration, no significant difficulties or misunderstandings were reported by participants, suggesting good acceptability of the instrument in this setting.

*Data Collection Procedure*. Data collection was conducted in anonymized form using printed questionnaires distributed by the research coordinator. Participants completed the forms during work breaks or after their shifts. All questionnaires were digitized for analysis. Although data collection began after the acute COVID-19 period, it is likely that the prolonged psychological and organizational stress generated by the pandemic continued to impact the healthcare workforce, particularly in under-resourced chronic care hospitals.

*Statistical Analysis*. All statistical analyses were performed using MedCalc (version 22.0). Descriptive statistics were used to summarize demographic and clinical variables. Continuous variables were expressed as means ± standard deviations (SD), and categorical variables as frequencies and percentages. Correlational analysis (Pearson and Spearman) was conducted to assess relationships between burnout dimensions, moral injury scores, ethical conflict frequency, and perceived support. Group differences (e.g., function, experience level) were tested using *t*-tests and one-way ANOVA. Multivariate linear and logistic regression analyses were used to identify predictors of high moral injury scores. The significance threshold was set at *p* < 0.05. For the logistic regression model, a median split of MISS-HP scores was used to dichotomize the outcome variable, given the absence of validated clinical cutoff points for this scale in the Romanian context. While we acknowledge that dichotomization can reduce statistical power and granularity, this approach facilitated a clearer interpretive framework for identifying high-risk groups within the sample. Continuous analyses were also explored in parallel but are not reported here due to lack of statistical significance and small sample size. Future research using larger samples and validated clinical thresholds is warranted.

Given the unbalanced gender distribution, additional subgroup comparisons based on sex were conducted using independent samples *t*-tests for continuous variables and chi-square tests for categorical outcomes where applicable. However, due to the limited number of male participants, the statistical power for gender-based inferences was low, and results were interpreted with caution.

## 3. Results

The final sample included 62 healthcare workers employed at the Chronic Diseases Hospital of Sebiș: 6 physicians (9.7%), 34 nurses (54.8%), and 22 auxiliary staff including nurse aides and orderlies (35.5%). The average age was 40 years (range 25–62), with a predominance of female participants (75%), which reflects the real gender structure of the hospital workforce. Professional experience ranged from 1 to 35 years, with a mean of 12.3 years. In terms of psychological outcomes, the mean emotional exhaustion (MBI-EE) score was 26.1, while moral injury (MISS-HP) scores averaged 5.3 out of 10. The majority of respondents reported moderate to high levels of perceived stress (mean = 6.8) and only moderate levels of perceived managerial support (mean = 2.9).

Burnout was assessed based on the Emotional Exhaustion (EE) subscale of the Maslach Burnout Inventory. A score of 27 or above was used to indicate high emotional exhaustion. Overall, 45.2% of the participants (28 out of 62) met criteria for high burnout. When analyzed by professional category, nurses showed the highest prevalence of burnout (50%), followed by auxiliary staff (45.5%) and physicians (33.3%).

Moral injury scores, assessed using the MISS-HP scale, revealed a moderate to high level of ethical distress across all professional categories. The mean moral injury score was 5.3 (SD = 1.4) on a scale from 1 to 10, indicating the presence of recurrent moral conflict and perceived ethical compromise.

**Figure 1:**
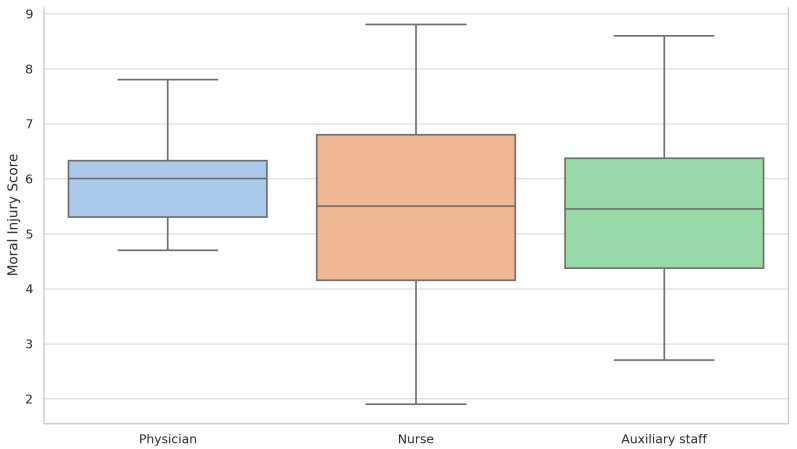
Moral injury scores by professional role.

As shown in Figure 1, nurses and auxiliary staff exhibited higher moral injury scores than physicians. Nurses reported a median score of 5.6, while auxiliary staff had a median of 5.4, both higher than physicians (median 4.7).

A more detailed comparison between professional categories is provided in Table 1, which summarizes mean scores for emotional exhaustion and moral injury, as well as the percentage of individuals above the clinical thresholds. Nurses reported both the highest average burnout and the highest proportion of staff with high moral injury.

**Figure 2:**
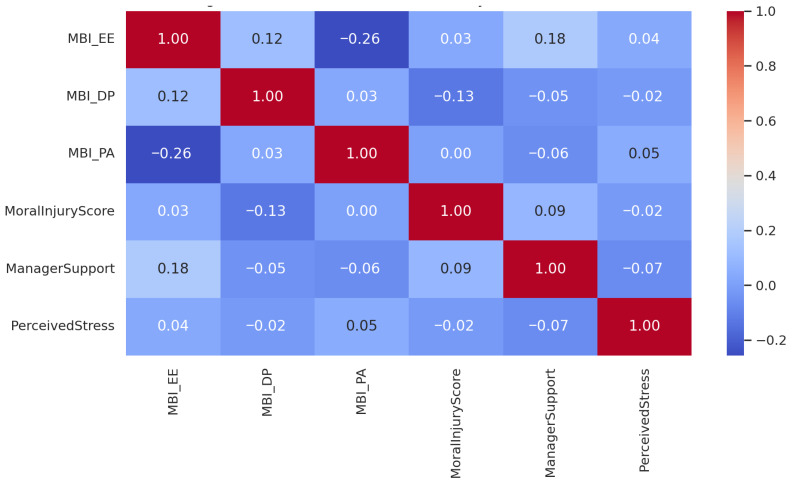
Pearson correlation matrix between key psychosocial variables: Emotional Exhaustion (MBI-EE), Depersonalization (MBI-DP), Personal Accomplishment (MBI-PA), Moral Injury Score, Managerial Support, and Perceived Stress. The color gradient represents the strength and direction of the correlation, from negative (blue) to positive (red), with values ranging from −1.0 to +1.0.

To explore the interrelations among key psychosocial variables, we conducted a Pearson correlation analysis between burnout dimensions (MBI subscales), moral injury, perceived managerial support, and perceived stress. The results are presented in Figure 2.

Figure 2 presents the Pearson correlation matrix for the main psychosocial variables. As shown, correlations between emotional exhaustion (MBI-EE), moral injury, managerial support, and perceived stress were generally weak and did not exceed r = 0.18 in any direction. The most notable findings were a small positive correlation between emotional exhaustion and perceived managerial support (r = 0.18), and a modest negative correlation between personal accomplishment (MBI-PA) and emotional exhaustion (r = −0.26), which is consistent with theoretical expectations. Importantly, moral injury was only weakly associated with any of the burnout subscales (EE: r = 0.03; DP: r = −0.13), suggesting that moral injury may not be strongly associated with general psychological strain in this sample. However, given the limited statistical power, this absence of correlation should be interpreted with caution and does not preclude a potential relationship in larger or differently structured populations.

To further examine whether burnout, perceived stress, or institutional support predict high levels of moral injury, a logistic regression analysis was conducted. The model included emotional exhaustion (MBI-EE), perceived managerial support, and perceived stress as independent variables, with high moral injury (MISS-HP score above median) as the outcome. The results indicated no statistically significant predictors: MBI-EE (β = −0.027, *p* = 0.246), managerial support (β = 0.124, *p* = 0.526), and perceived stress (β = 0.038, *p* = 0.744). These findings suggest that moral injury may be influenced by systemic ethical or organizational factors beyond individual burnout or stress levels.

**Figure 3:**
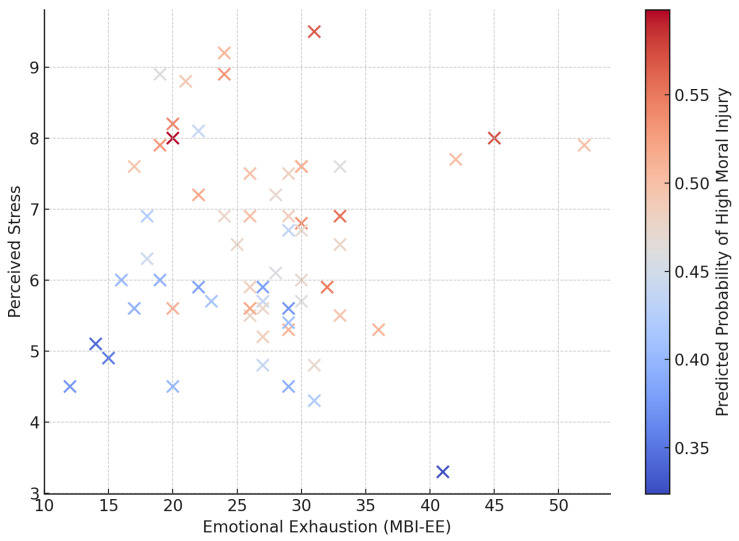
Predicted probability surface for high moral injury based on emotional exhaustion (MBI-EE) and perceived stress. The plot displays a logistic regression model output, where warmer colors indicate higher predicted probability of scoring above the median MISS-HP value. The surface illustrates how combinations of higher emotional exhaustion and stress may correspond to increased risk, although no strong linear interaction was found. This visual is intended to illustrate model-based risk estimates in two dimensions for explanatory purposes.

Figure 3 illustrates the predicted probability surface for high moral injury based on emotional exhaustion and perceived stress. As shown, although areas with higher MBI-EE and stress tended to correspond to slightly increased predicted risk, no clear threshold or gradient emerged. This visual pattern further supports the absence of a strong predictive relationship and underscores the need for more nuanced models that can separate the overlap and distinctions between moral injury and burnout.

## 4. Discussion

This study assessed the prevalence of burnout and moral injury among healthcare workers in a Romanian chronic care hospital and explored the relationships between these outcomes and psychosocial variables such as perceived stress and managerial support. Although nearly half of the participants exhibited high emotional exhaustion, and average moral injury scores were moderate to high, our regression model did not reveal statistically significant associations between these variables.

The unique characteristics of chronic care hospital extended patient-provider relationships, emotional attachment, resource lack, and frequent ethical conflicts, create a workplace dynamic that differs significantly from general or acute care facilities. By focusing on this context, the study provides insight into an institutional setting often overlooked in broader moral injury research. The findings highlight the necessity for ethical support frameworks tailored to long-term care environments, particularly in low-resource systems.

These findings suggest that moral injury, although conceptually linked to burnout, may arise from distinct systemic and ethical factors rather than from emotional exhaustion alone. This observation is consistent with the evolving understanding of moral injury as a complex, multidimensional phenomenon rooted in ethical transgressions, perceived betrayal, or inability to act in alignment with professional values due to institutional constraints [8,9]. Unlike burnout, which can often be mitigated through individual-level interventions such as workload adjustments or resilience training, moral injury requires structural changes in healthcare delivery, ethical climate, and leadership accountability [23].

Our findings align with emerging research showing that burnout and moral injury, although related, stem from partially distinct sources. For instance, Purcell et al. (2024) demonstrated that moral injury in hospital staff was more strongly associated with perceived ethical climate and institutional betrayal than with workload or emotional exhaustion alone [24]. Similarly, a multinational study by Gómez-Ochoa et al. (2022) highlighted that inadequate ethical support structures were a key predictor of moral distress during and after the COVID-19 pandemic [25]. These findings support the hypothesis that moral injury requires organizational and moral infrastructure interventions, rather than individual-level stress reduction strategies. Moreover, transdiagnostic processes—such as psychological inflexibility, maladaptive coping, and moral dissonance—are increasingly recognized as shared vulnerability factors across occupational mental health outcomes [15]. These mechanisms may explain why burnout and moral injury can coexist yet diverge in their clinical manifestations and response to interventions.

The context of data collection is particularly important in interpreting our results. The study commenced in October 2022, shortly after the acute phases of the COVID-19 pandemic. Although we did not include specific items measuring pandemic-related exposure or distress, it is plausible that the long-term effects of that period—such as emotional fatigue, organizational disillusionment, and ethical conflict—persisted among staff. Prior studies have shown that moral distress can linger after acute crises, especially in settings with limited institutional recovery mechanisms [10,15]. Nevertheless, we acknowledge that attributing moral injury symptoms to pandemic-related factors in our study remains speculative and should be interpreted as contextual background rather than causal inference. Prolonged exposure to triage-related decisions, high patient mortality, and institutional overload during the pandemic may have left a residual imprint on staff, contributing to a form of “delayed moral injury” that is not fully explained by conventional burnout indicators [26].

Moreover, the setting of the study—a chronic care hospital with limited staffing, resources, and visibility—further exacerbates the ethical vulnerabilities of frontline workers. Chronic care environments are known for high emotional labor, long-term patient relationships, and exposure to end-of-life dilemmas, all of which amplify ethical strain and increase the risk of moral fatigue [27,28].

Although the correlation between burnout and moral injury was not statistically significant in this sample, the high prevalence of both conditions suggests the existence of shared organizational risk factors. This finding aligns with reports from other European studies, such as one conducted by Vinckers et al. (2025) [29], which found that moral distress was prevalent in long-term care nurses regardless of individual burnout levels. Despite growing international literature, limited research has explored the inter-section between burnout and moral injury in Eastern European healthcare systems. In Romania, where public hospitals face chronic underfunding, staff shortages, and unclear ethical frameworks, chronic care institutions represent high-risk environments for both syndromes. This pattern is mirrored in other European contexts: in Italy, Barello et al. (2020) [6] found high levels of burnout and psycho-somatic symptoms among healthcare workers strained by resource scarcity, ethical dilemmas, and overwhelming job demands

These results reinforce the hypothesis that moral injury is not merely an extension of burnout but may emerge from unresolved ethical tensions, hierarchical imbalance, or lack of institutional responsiveness. In this context, chronic care units—often overlooked in national policy debates—may act as ethical “pressure cookers,” where the prolonged care of vulnerable patients increases exposure to moral dissonance.

The persistence of moral injury symptoms in post-pandemic healthcare environments may also be influenced by individual-level psychological processes. One such process is psychological inflexibility, a central construct in Acceptance and Commitment Therapy (ACT) models, which refers to the inability to remain in contact with present experiences and values when faced with psychological distress. Recent findings by Di Gesto et al. (2025) [15] indicate that psychological inflexibility fully mediates the relationship between difficulties in emotion regulation and perceived stress in ICU nurses exposed to ethical strain. Their study also found that longer clinical experience moderated this link, suggesting that both personal coping styles and contextual exposure shape vulnerability to moral injury [15]. Although our study did not directly assess these variables, such evidence highlights the relevance of transdiagnostic psychological mechanisms in mediating the impact of institutional constraints on moral well-being. Future research in chronic care environments should consider incorporating these variables to better understand staff responses to persistent ethical challenges. Another emerging transdiagnostic mechanism relevant to occupational resilience is self-compassion—the ability to relate to one’s own distress with kindness and balanced awareness [30]. Studies in healthcare workers have shown that higher levels of self-compassion are associated with lower rates of burnout and moral distress, as well as better emotional regulation and ethical engagement. Self-compassion may serve as a buffer against moral dissonance by fostering reflective coping rather than guilt-based rumination [31,32]. Future interventions in chronic care institutions could benefit from integrating both structural reforms and individual-focused strategies, such as compassion-based training, to reduce cumulative ethical strain.

Despite efforts to reduce burnout, systemic challenges still fuel moral injury in healthcare professionals. As defined by Linzer and Poplau (2021) [33], moral injury occurs when clinicians are forced to participate in or witness actions that violate their deeply held moral beliefs. This construct is closely correlated with burnout, yet extends beyond emotional exhaustion, reflecting deeper ethical and existential conflicts within healthcare systems. Their findings underscore the need for bold organizational changes, emphasizing that addressing structural causes—rather than focusing solely on individual resilience—is essential to reduce both burnout and moral injury in medical staff.

The practical implications are substantial. Beyond psychological counseling, chronic care institutions should consider implementing ethics consultation services, peer support groups, and protocols for debriefing after ethically charged situations. Training mid-level managers in ethical leadership and communication can further buffer staff against cumulative moral stress [34,35].

This study has some limitations. First, the data were collected from a single institution, which may limit generalizability. However, this chronic care hospital represents a specific type of healthcare environment that is structurally and ethically distinct from larger acute care centers. The prolonged exposure to chronic suffering, limited institutional support, and cumulative moral stress encountered in such facilities offer a valuable perspective on underexplored dimensions of staff vulnerability. Rather than a limitation per se, the study setting can be regarded as a strength in terms of internal validity and contextual relevance.

Second, the reliance on self-reported scales may introduce bias. Finally, we did not assess protective variables such as professional identity, spirituality, or resilience, which may moderate the experience of moral injury. Despite these constraints, the study offers valuable insight into the ethical landscape of Romanian chronic care and highlights areas for urgent organizational reform. Additional limitations include the relatively small sample size, which may reduce the statistical power to detect significant associations—particularly in regression analyses or subgroup comparisons. The predominantly female composition of the sample, although reflective of the gender distribution in Romanian chronic care facilities, may also limit the generalizability of findings to more gender-balanced or male-dominated healthcare environments. Furthermore, the cross-sectional design precludes causal inference, and the use of self-reported measures may introduce response bias or social desirability effects. Lastly, the study did not include measures of individual psychological traits, such as coping styles or resilience, which could mediate the relationship between workplace factors and moral injury. Nevertheless, this study was not designed to estimate prevalence rates at the population level, but rather to explore psychological dynamics within a defined institutional setting. As such, it can be regarded as a pilot investigation that provides groundwork for larger-scale studies. This is particularly relevant given the lack of empirical data on moral injury in Eastern European chronic care systems. Despite the small sample, the inclusion of nearly the entire clinical staff from the hospital improves internal validity and enhances the relevance of the findings for this context. While we did not conduct an a priori power analysis, we recognize that the inclusion of multiple predictors in regression models with a relatively small sample size increases the risk of type II error. The absence of statistically significant associations between variables—such as burnout and moral injury—should therefore be interpreted with caution. These null results may reflect insufficient statistical power rather than true absence of relationships. As such, our findings should be considered hypothesis-generating, requiring confirmation in future studies with larger, adequately powered samples.

Another limitation is that our regression models included only three predictors (emotional exhaustion, perceived stress, and managerial support) and did not adjust for potentially important confounders such as gender, professional role, or years of experience. The decision to limit the number of predictors was guided by the small sample size and the need to avoid overfitting. However, we recognize that these demographic and occupational variables may influence both perceived stress and moral injury and should be incorporated in future multivariate models with greater statistical power.

## 5. Conclusions

Burnout and moral injury are prevalent among healthcare workers in Romanian chronic care settings, particularly among nurses and long-tenured staff. While no direct statistical link was found between burnout, stress, and moral injury, the presence of moderate-to-severe moral distress highlights deeper systemic issues. The study underscores the need for organizational interventions that address ethical climate, institutional support, and the lingering impact of the COVID-19 pandemic. Moral injury should be recognized as a distinct phenomenon requiring targeted strategies beyond burnout prevention. Future research should explore longitudinal patterns and institutional factors to better inform prevention and recovery efforts in vulnerable healthcare environments.

## Figures and Tables

**Table 1:** Summary of psychological outcomes by professional role. Values represent the mean and standard deviation (SD) of Emotional Exhaustion (MBI-EE) and Moral Injury (MISS-HP) scores, along with the proportion of participants exceeding established thresholds (MBI-EE ≥ 27; MISS-HP > sample median).

Professional Role	n	MBI_EE (Mean ± SD)	High EE (%)	Moral Injury (Mean ± SD)	High Moral Injury (%)
Physicians	6	24.7 ± 6.1	33.3%	4.9 ± 1.2	50.0%
Nurses	34	26.8 ± 7.4	50.0%	5.5 ± 1.4	55.9%
Auxiliary Staff	22	25.9 ± 6.6	45.5%	5.4 ± 1.5	50.0%

## Data Availability

The data presented in this study are available on request from the corresponding author.
